# Emergence and Transmission of Plasmid-Mediated Mobile Colistin Resistance Gene *mcr-10* in Humans and Companion Animals

**DOI:** 10.1128/spectrum.02097-22

**Published:** 2022-08-24

**Authors:** Yi Yin, Lihao Qiu, Guizhen Wang, Zhimin Guo, Zhiqiang Wang, Jiazhang Qiu, Ruichao Li

**Affiliations:** a Jiangsu Co-Innovation Center for Prevention and Control of Important Animal Infectious Diseases and Zoonoses, College of Veterinary Medicine, Yangzhou University, Yangzhou, Jiangsu, People’s Republic of China; b State Key Laboratory for Zoonotic Diseases, College of Veterinary Medicine, Jilin University, Changchun, People’s Republic of China; c College of Food Engineering, Jilin Engineering Normal University, Changchun, People’s Republic of China; d Department of Clinical Laboratory, The First Hospital of Jilin University, Changchun, People’s Republic of China; e Institute of Comparative Medicine, Yangzhou University, Yangzhou, Jiangsu, People’s Republic of China; INTHERES

**Keywords:** *mcr-10*, *Enterobacter cloacae* complex, companion animals, genetic analysis

## Abstract

Mobile colistin resistance (*mcr*) genes mediated by plasmids have widely disseminated throughout the world. Recently, 10 *mcr* genes (*mcr-1* to *mcr-10*) and a large number of variants have been identified in more than 60 countries. However, only a few instances of Enterobacter cloacae complex (ECC) bearing *mcr-10* from animal origin have been reported globally. The aim of this study was to fill a knowledge gap in *mcr-10*-positive ECC of animal origin and analyze the potential transmission trend and different characteristics between human and companion animal isolates. The *mcr-10* gene was identified on a self-transmissible plasmid in the human isolate and non-transmissible plasmids in other three animal strains. *mcr-10* was adjacent to a XerC-type tyrosine recombinase-gene, and various insertion sequences were located on the downstream of core conservative structure *xerC-mcr-10*, thus indicating this region might be a candidate for insertions of mobile genetic elements and *mcr-10* might be mobilized by IS-mediated mechanisms. Moreover, phylogenetic analysis found that *mcr*-*10*-positive isolates were mainly distributed in the clade of Enterobacter *roggenkampii*, exhibiting significant species specificity. These findings indicated that *mcr-10* has emerged among Enterobacter spp. within humans and companion animals, highlighting that the importance of taking effective control measures to monitor the dissemination and evolution of *mcr* genes.

**IMPORTANCE** Colistin was considered as the last-resort drug against severe clinical infections caused by multidrug-resistant Gram-negative pathogens. Mobile colistin resistance (*mcr*) genes and its variants carried by plasmids have been reported in diverse niches in recent years, and yet few studies reported carriage of *mcr-10* in ECC strains of companion animal origin. How plasmid-borne *mcr-10* transmitted in opportunistic pathogens and different characteristics of *mcr-10*-bearing strains isolated from humans and companion animals are not well understood. In this study, we discovered *mcr-10*-harboring strains in multidrug-resistant ECC isolates of companion animal origin for the first time and conducted a comprehensive analysis of the genetic environment of *mcr-10* from multiple countries around the world, providing the potential basis for formulating control measures to slow down the spread of colistin resistance.

## INTRODUCTION

Among *Enterobacteriaceae* bacteria, the Enterobacter cloacae complex (ECC) was found to be associated with a high rate of infections resulting from *Enterobacteriaceae* in clinical settings (1, 2), including septicemia, lower respiratory tract infections, as well as skin and soft tissue infections. It has been demonstrated that ECC is an opportunistic organism that can survive in a wide range of hosts, such as humans, animals, and plants. Molecular and biochemical analyses of the ECC have uncovered genomic and genotypic heterogeneity, and it now comprises the following Enterobacter species: E. cloacae, E. asburiae, E. hormaechei, E. kobei, E. ludwigii, E. mori, and E. nimipressuralis (2), of which E. cloacae and *E.* hormaechei are the most commonly discovered in clinical specimens from humans and animals, respectively (3). In addition, carbapenemase genes such as *bla*_IMP_, *bla*_NDM_, *bla*_GIM_, or *bla*_KPC_ of plasmid origin have been identified in the ECC, not only creating the potential risk for transmission but also reducing the effectiveness of the carbapenems (4).

The emergence and spread of carbapenem-resistant *Enterobacteriaceae* (CRE) pathogens have caused a huge public health hazard globally (5). Colistin, a cationic polypeptide with antibacterial and biocidal activity, serves as a last line of defense against severe CRE infections (6). Nevertheless, the emergence and global distribution of colistin-resistant CRE significantly limit the use of colistin in clinical practice (7). Colistin resistance was present in 0.67% of the total *Enterobacteriaceae* isolates collected over a 4-year period, and the colistin resistance was higher in ECC (4.2%) than that in Escherichia coli and Klebsiella pneumoniae (0.5% and 0.4%, respectively) (2, 8). It was worth noting that the high colistin resistance with MIC up to 256 mg/L in ECC isolates was not linked with mobile colistin resistance (*mcr*) genes, speculating that other mechanisms mediate colistin resistance (8). In line with this, Xu et al. observed that the elimination of *mcr*-positive plasmid had no effect on the MIC value of colistin, further suggesting that there was no direct connection between resistance gene and resistance phenotype (9). Trimble, Michael J et al. found that chromosomal mutations in two-component systems (TCSs), *phoPQ* and *pmrAB* or their regulators like *mgrB*, might enhance the mechanisms that modify lipopolysaccharides (LPS), and subsequently increase the colistin MIC (10). Nevertheless, in contrast to Klebsiella spp. and E. coli, there is a greater number of random mutations in ECC, which has made it difficult to identify the specific amino acid substitutions conferring colistin resistance (11).

In addition to the chromosome-mediated colistin mechanism, the plasmid-carrying *mcr-1* gene was first discovered in porcine *Enterobacteraceae* in 2016 (12), and since then, *mcr* variants have been discovered in various species with different origins all over the world, showing that plasmid is equipped with flexible transmission ability through concerted activities within or between DNA molecules (13–15). Most recently, the discovery of novel *mcr-10* located on chromosomes or transmissible plasmids has attracted considerable attention. Human, hospital sewage water and food are the principal sources of such isolates (9, 11). In accordance with 'One Health', it is also crucial to monitor the distribution of *mcr* genes in animals, particularly in companion animals, since antimicrobial agents used in companion animals are often more closely related to ones used in humans (16, 17), thus antimicrobial resistance (AMR) risks should not be ignored. In certain studies, *mcr* genes have been identified in companion animals' populations of different species, such as various *mcr* variants in K. pneumoniae (18), *mcr-1* in E. coli (19), *mcr-8* in K. pneumoniae, *mcr-9* in E. hormaechei (20). It is evident from these data that companion animals play a significant role in the dissemination of the *mcr* gene. Moreover, the close relationship between companion animals and humans makes it possible for AMR bacteria to be transferred between two sides (21). Hence, it is essential to analyze the different characteristics of *mcr-10-*positive strains isolated from companion animals and humans, and shed light on the phylogenetic relationship of *mcr-10*-positive strains.

## RESULTS

### Identification of *mcr-10* in colistin-resistant E. cloacae complex.

In this study, a total of 4 *mcr-10*-bearing isolates were recovered from 294 feces samples (healthy cats and dogs) and 734 clinical strains from Jilin province, China. Species identification determined the *mcr-10*-harboring isolates as E. roggenkampii and E. hormaechei with average nucleotide identity (ANI) values above 98% (Table 1). Multilocus sequence type (MLST) analysis assigned the 4 strains to ST984, ST143, ST681, and ST965. The animal-derived strains, K475-2, K666 and K528 were resistant to ampicillin (>128 mg/L), ceftazidime (16-32 mg/L), colistin (≥8 mg/L), but susceptible to tigecycline (≤0.25 mg/L) and meropenem (≤0.25 mg/L), presenting a similar susceptibility profile. In contrast, the human-derived Ek140 was susceptible to most tested drugs except for colistin (8 mg/L), ceftazidime (32 mg/L) and ampicillin (>128 mg/L).

**TABLE 1:** Antimicrobial susceptibility testing and resistance genotypes of 4 *mcr-10*-positive isolates in this study

			Source	MIC(mg/L)^*a*^									
Strain	Species	STs		TIG	AMP	CAZ	CIP	KAN	MEM	CL	AMK	Plasmid type	Resistance genes
K475-2	Enterobacter *roggenkampii*	984	Healthy cat	≤0.25	>128	32	16	>128	≤0.25	128	8	IncHI2A, IncHI2, **IncFIB, IncFII**^*b*^	*bla*_MIR-15_*, fosA, oqxA10, oqxB9, aph(3′)-Ia, floR, aph(6)-Id, tet*(A)*, sul3, lnu*(F)*, aadA22, aac(3)-IId, bla_LAP-2_, bla*_TEM-1_*, bla*_CTX-M-55_*, qnrS1, mph*(A) *arr-2, dfrA14,* ***mcr-10.1***
K666	Enterobacter *roggenkampii*	143	Healthy cat	≤0.25	>128	16	4	8	≤0.25	8	8	**IncFII**, IncFIB	*bla*_MIR-11_*, fosA, oqxA10, oqxB9,* ***bla*_TEM-1_*, bla*_LAP-2_*, qnrS1, mcr-10.1***
K528	Enterobacter hormaechei	681	Healthy dog	≤0.25	>128	16	4	1	≤0.25	8	2	**IncFII, IncFIB**	*bla*_ACT-25_*, fosA, oqxB9, oqxA9,* ***catA1, aadA1, sul2, aph(3″)-Ib, aph(6)-Id, bla*_TEM-1_*, bla*_SHV-12_*, qnrB1, dfrA14, tet*(A)*, mcr-10.1***
Ek140	Enterobacter *roggenkampii*	965	Human pus	≤0.25	>128	32	≤0.25	1	≤0.25	8	2	**IncFII, IncFIB**	*bla*_MIR-10_*, oqxA10, oqxB20,* ***mcr-10.1***

a TIG, tigecycline; AMP, ampicillin; CAZ, ceftazidime; CIP, ciprofloxacin; KAN, kanamycin; MEM, meropenem; CL, colistin; AMK, amikacin.

b The Inc type and resistance genes of *mcr-10-*bearing plasmids were highlighted in bold fonts.

The chromosome of 4 ECC isolates shared similar antimicrobial resistance genes including *bla*_MIR_ or *bla*_ACT_ (a chromosomal *ampC* gene intrinsic to E. cloacae complex mediating resistance to almost all β-lactam antibiotics) and *oqxAB* (an efflux pump that confers resistance to phenicol and quinolone [22]). Additionally, the plasmids of 2 isolates recovered from companion animals carried multidrug resistance genes (*aph*(*3′*)*-Ia*, *aac*(*3*)*-IId*, *bla*_LAP-2_, *bla*_TEM-1_, *bla*_CTX-M-55,_ *aph*(*6*)*-Id*, *aadA22*, *tet*(A), *sul3*, *dfrA14*, etc.), while pEk140_mcr-10 and pK475-2_mcr-10 did not carry any other resistance genes except *mcr-10*.

### Genomic features of *mcr-10-*positive strains.

A bacterial conjugation assay showed that *mcr-10* carried by Ek140 was self-transmissible, and the transfer frequency was 2 × 10^−8^ per recipient cell. The transconjugant strain was confirmed to harbor *mcr-10* by PCR and resistant to colistin with MIC 4 mg/L. Despite repeated attempts of conjugation experiments, transconjugants for the remaining 3 strains were not obtained, suggesting that they were not self-transmissible.

To determine the location of *mcr-10*, 4 colistin-resistant ECC isolates were subjected to short-read and long-read whole-genome sequencing. Replicon typing showed that they belonged to IncFII-FIB (pK475-2_mcr-10, pK528_mcr-10 and pEk140_mcr-10) and IncFII (pK666_mcr-10). Human origin pK140_mcr-10 shared 31%–36% coverage with 3 animal derived plasmids by pairwise comparison of the plasmid backbone sequences, indicating they were dramatically different from each other and without direct transmission relationship (Table S3). Comparing the 4 plasmids with the whole 31 *mcr-10*-harboring completely sequenced plasmids in GenBank (Fig. S1A), we found that not only the structure *xerC-mcr-10* was conserved in investigated plasmids but also the Inc type of *mcr-10*-positive plasmids mostly belonged to IncF (IncFII, IncFIA and IncFIB), with the exception of one plasmid co-harbored IncFIB and IncR (Table 2). Furthermore, *mcr-10*-bearing plasmids were found in a variety of genus, including Enterobacter, Klebsiella, Riemerella, Escherichia and Citrobacter, with Enterobacter spp. being the most common host (23/31, 74.2%), and the number of K. pneumoniae strains being second only to Enterobacter spp., speculating that *mcr-10*-positive plasmids were of genus and IncF type specificity.

**TABLE 2:** Basic information of plasmids harboring *mcr-10* in the NCBI database

Plasmid	Host	year	Country	Source	Length (bp)	Inc type	Accession no.
pMCR10_090065	Enterobacter *roggenkampii*	2016	China	Unknown, human	71775	IncFIA	CP045065
pYK16-mcr-10	Enterobacter *roggenkampii*	2019	China	Chicken	117855	IncFII	MT468575
pEcl_20_981	Enterobacter *roggenkampii*	2019	China	Medical waste water	161986	IncFII, IncFIB	CP048651
pSTW0522-66-1	Enterobacter *roggenkampii*	2018	Japan	Sewage water	324199	IncFII, IncFIB	AP022466
pN260-2	Enterobacter *roggenkampii*	2109	Japan	Unknown, human	244996	IncFIB, IncR	AP023449
p13840_1	Enterobacter *roggenkampii*	2013	China	Sputum, human	94721	IncFII, IncFIB	CP083820
pEr983-1	Enterobacter *roggenkampii*	N.A.	China	Sewage water	100102	IncFIB	CP060738
pRHBSTW-00399_2	Enterobacter cloacae	2017	United Kingdom	Sewage water	137623	IncFII, IncFIA	CP056561
pEN37S	Enterobacter cloacae	2015	Japan	Dogs	70277	IncFIB	AP024497
pAVS0889-b	Enterobacter cloacae	2021	Switzerland	River water	90827	IncFIB	CP092044
pEC27-2	Enterobacter cloacae	2010	Viet Nam	Urine, huamn	84602	IncFIA	CP020091
pSL12517-mcr10.1	Enterobacter cloacae	N.A.	N.A.	N.A.	58151	IncFIA	MW048777
p12961_1	Enterobacter cloacae	2012	China	Urine, huamn	90256	IncFIA	CP083822
pRHBSTW-01009_2	Enterobacter asburiae	2107	United Kingdom	Sewage water	70650	IncFIA, IncFIB	CP056127
pECLA	Enterobacter asburiae	2019	China	Urine, huamn	159417	IncFII, IncFIB	CP093155
p161373_1	Enterobacter asburiae	2016	China	Sputum, human	154756	IncFII, IncFIB	CP083816
pEL-ars1	Enterobacter ludwigii	2109	China	Tailing mud	145699	IncFII, IncFIB	CP094842
p11894_1	Enterobacter ludwigii	2011	China	Throat swab, huamn	131857	IncFII, IncFIB	CP083825
pSTW0522-51-1	Enterobacter kobei	2018	Japan	Sewage water	159829	IncFII, IncFIA	AP022432
p11778_1	Enterobacter kobei	2011	China	Secretion, human	130391	IncFII, IncFIB	CP083829
pECC59-2	Enterobacter hormaechei	2017	China	Broncho-alveolar lavage, human	64293	IncFIA	CP080472
pRHBSTW-00175_3	Enterobacter sp.	2017	United Kingdom	Freshwater	68715	IncFIB	CP055932
pECC18A13-1	Enterobacter sp.	2018	Japan	River water	150509	IncFII	AP019635
pKP46-mcr10	Klebsiella pneumoniae	2020	China	Feces, chicken	186056	IncFII, IncFIB	CP088124
pKP57-mcr10	Klebsiella pneumoniae	2020	China	Feces, chicken	186040	IncFII, IncFIB	CP088129
p5	Klebsiella pneumoniae	2018	Sierra Leone	N.A.	120029	IncFII, IncFIA	LR890193
pKqs_SB610_4	Klebsiella quasipneumoniae	2000	Netherlands	Water	124980	IncFII, IncFIB	CP084774
pNUITM-VR1_2 DNA	Raoultella ornithinolytica	2021	Viet Nam	N.A.	261835	IncFII, IncFIB	AP025011
unnamed1	Raoultella ornithinolytica	2015	Canada	Rectal, human	231294	IncFII, IncFIB	CP023893
pEC81-mcr10	Escherichia coli	2020	China	Feces, human	62662	IncFII, IncFIA	CP088133
pOZ172	Citrobacter freundii	1998	China	Leg ulcer, human	127005	IncFII, IncFIB	CP016763

**FIG 1:**
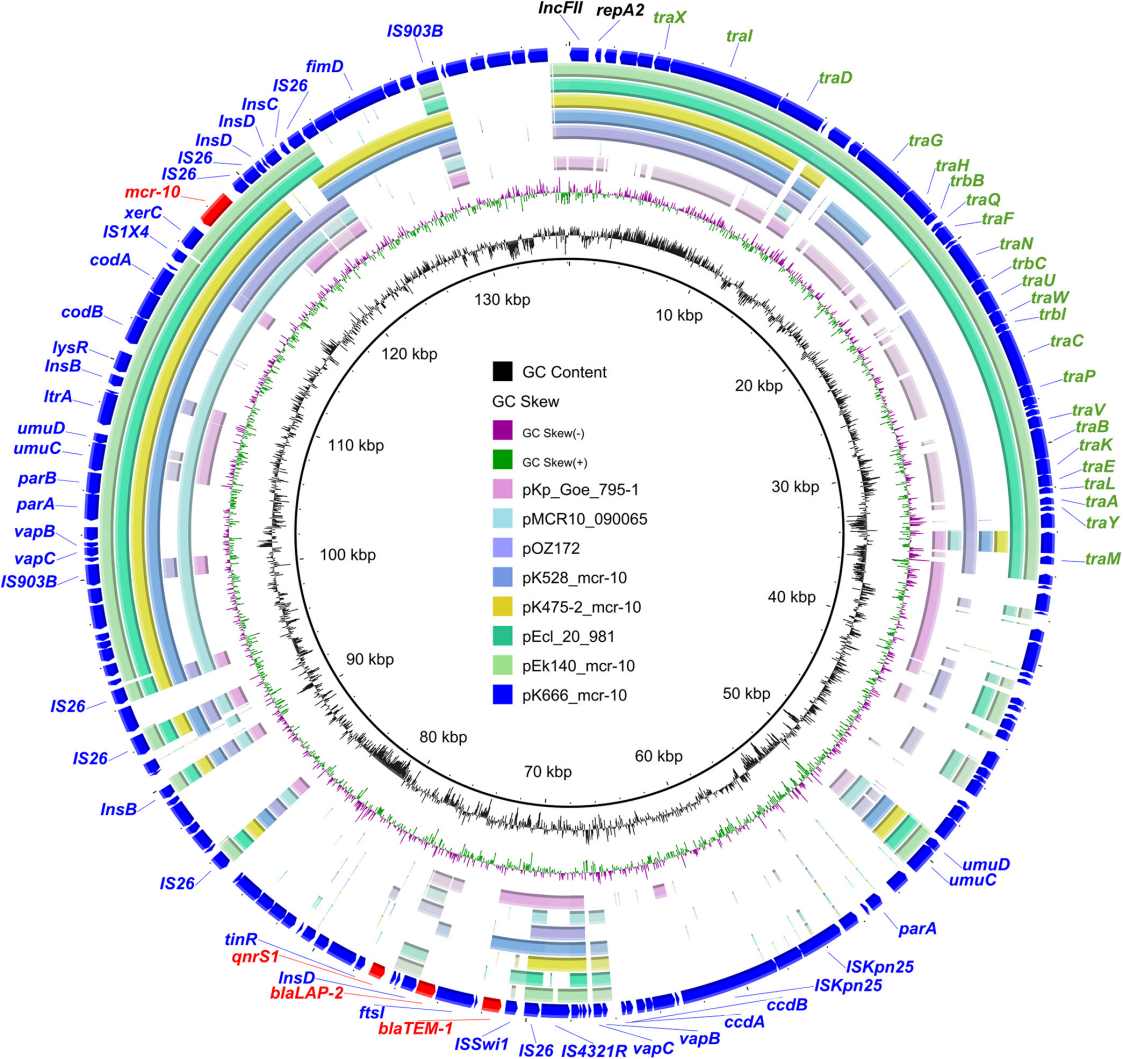
Genetic environment of *mcr-10*-positive plasmids. GC skew and GC content are indicated from the inside out. The arrows represent the positions and transcriptional directions of the ORFs. Genes are differentiated by colors.

The human origin plasmid, designed as pEk140_mcr-10, encoded 650 ORFs with a GC content of 53.6% (Fig. 1 and Fig. S1B). Comparison of pEk140_mcr-10 with other plasmids revealed that it was 50% coverage and 99.2% identity to the plasmid pOZ172 (CP016763) of Citrobacter freundii strain B38, the first discovered plasmid harboring *mcr-10*, obtained from Sun Yat-sen Memorial Hospital in Guangzhou, December 1998. BLASTn of both pK666_mcr-10 and pK475-2_mcr-10 of animal origin against nr database showed that the high homology was an *mcr-10*-bearing IncFII-FIB plasmid pEcl_20_981 detected in an E. roggenkampii, which was isolated from medical wastewater in Shenzhen in 2019, with 98.4%, 99.6% identity and 56%, 71% coverage, respectively. Furthermore, pK528_mcr-10 showed 58% coverage and 99.9% identity with pKp_Goe_795-1, which was isolated from K. pneumoniae belonging to ST15. Recently, this pathogen was identified as the cause of several nosocomial infections in Asia, Europe, Africa, and Brazil (23).

### The genetic contexts of *mcr-10*.

The colistin resistance gene *mcr-10* located in different plasmids of 4 ECC strains was adjacent to a XerC-type tyrosine recombinase-gene, designed as *xerC.* XerC-type tyrosine recombinases containing a C-terminal DNA breaking-rejoining catalytic domain, known to be involved in mediating horizontal transfer of antimicrobial resistance genes within hosts via site-specific recombination. Furthermore, an IS*Ecp36* was present at downstream of *mcr-10* in pEk140_mcr-10 (Fig. S2), sharing a similar structure with pEcl_20_981, however, was different with the first reported *mcr-10*-positive plasmid, pMCR10_090065 (accession number CP045065), in which 2 copies of truncated IS*903B* were located at the upstream and downstream of *mcr-10*, forming a composite transposon. Analysis of genomic data of pK666_mcr-10 demonstrated that *mcr-10* was bracketed by 2 insertion sequences (IS). Upstream of *mcr-10*, truncated IS*1X4* (IS*1* family) was identified and another IS, namely, IS*26* (IS*6* family) was located downstream.

**FIG 2:**
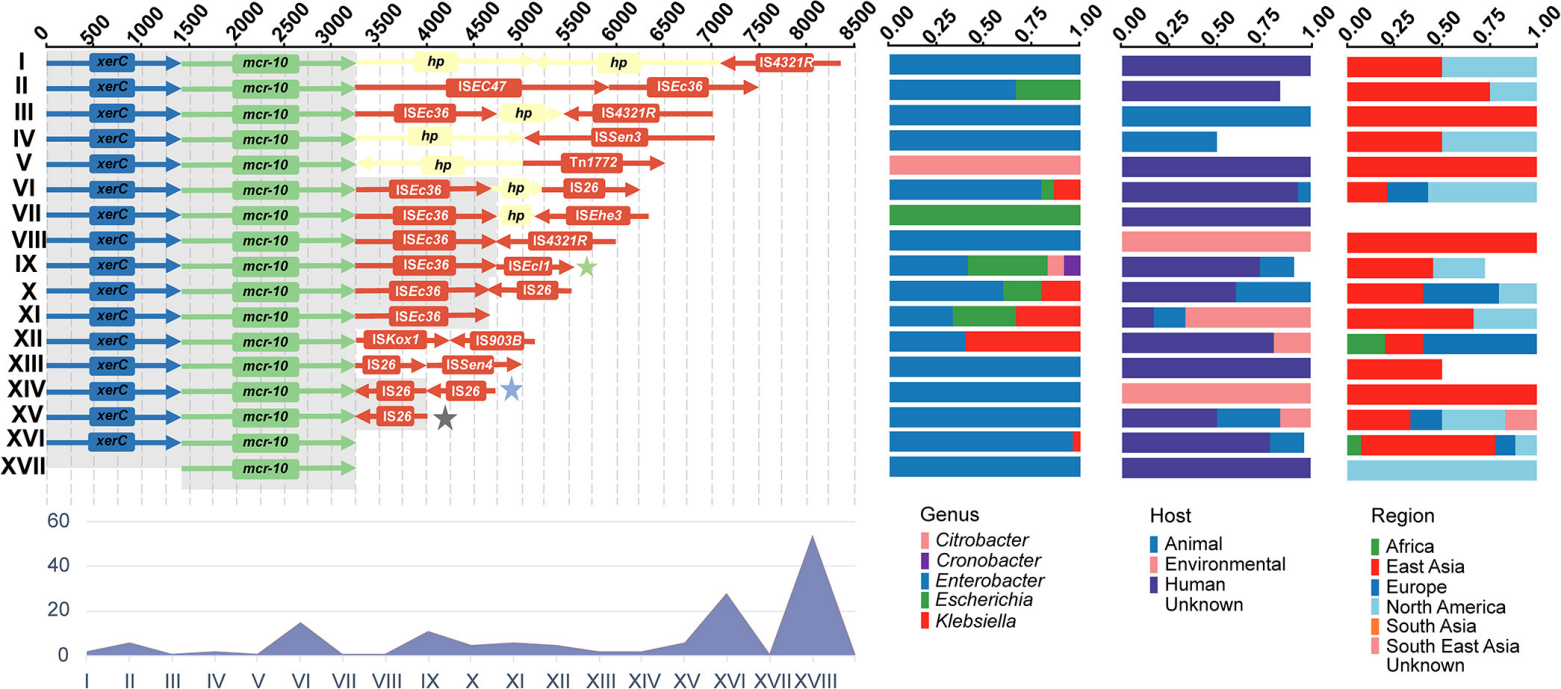
Structural variants downstream of *xerC-mcr-10* of 145 isolates (in GenBank, see Table S4 in the supplemental material) and 4 strains in this study. On the left of the figure are the most prevalent structural variations supplied by the Prokka and Roary pipelines. The gray shading indicates the homologous areas between structural variations. Some structural variations and branches were purposefully shortened, despite the fact that their contigs were of appropriate size or greater. A total of 16 types were divided according the downstream structures of *mcr-10* and the number of each structure was demonstrated in the below figure. Of which, type XVIII was identified as uncertain structure due to short contig length. Three structures marked with different colors of pentagrams indicated the genetic environment of 4 strains (green, pEk140_mcr-10; blue, pK666_mcr-10 and pK528_mcr-10; gray, pK475-2_mcr-10). The right-hand side shows the distribution of genus, host, and geographical regions of samples that belong to each common structural variant.

To further detect the mobilization mechanism of *mcr-10*, a total of 145 matches were identified in GenBank (Fig. 2 and Table S4). Analysis of the genetic environment in 145 genomes showed that the structure *xerC-mcr-10* was conserved (141/145 97.2%) and the downstream flanking region exhibited more IS structural diversification than the upstream region, suggesting that this area may be a candidate for mobile genetic element insertions. The complete genetic structure could not be determined in nearly one-third of genomes (54/145 37.2%) since the draft genomes cannot cover the regions. Many of 45 analyzed contigs contained IS*Ec36* and IS*26*, which were the most potential mobile elements that mediate the transmission of *mcr-10*. Furthermore, *mcr-10* was seen in contigs mainly from Enterobacter spp. (72/95 75.8%), in line with the host distribution of *mcr-10*-positive plasmids. Human (67/95 70.5%) and environmental (15/95 15.8%) samples were the most common sources of *mcr-10*-bearing strains, indicating the wide spread of *mcr-10* around the world, especially in East Asia and North America.

### Phylogenetic analysis of *mcr-10-*bearing isolates.

**FIG 3:**
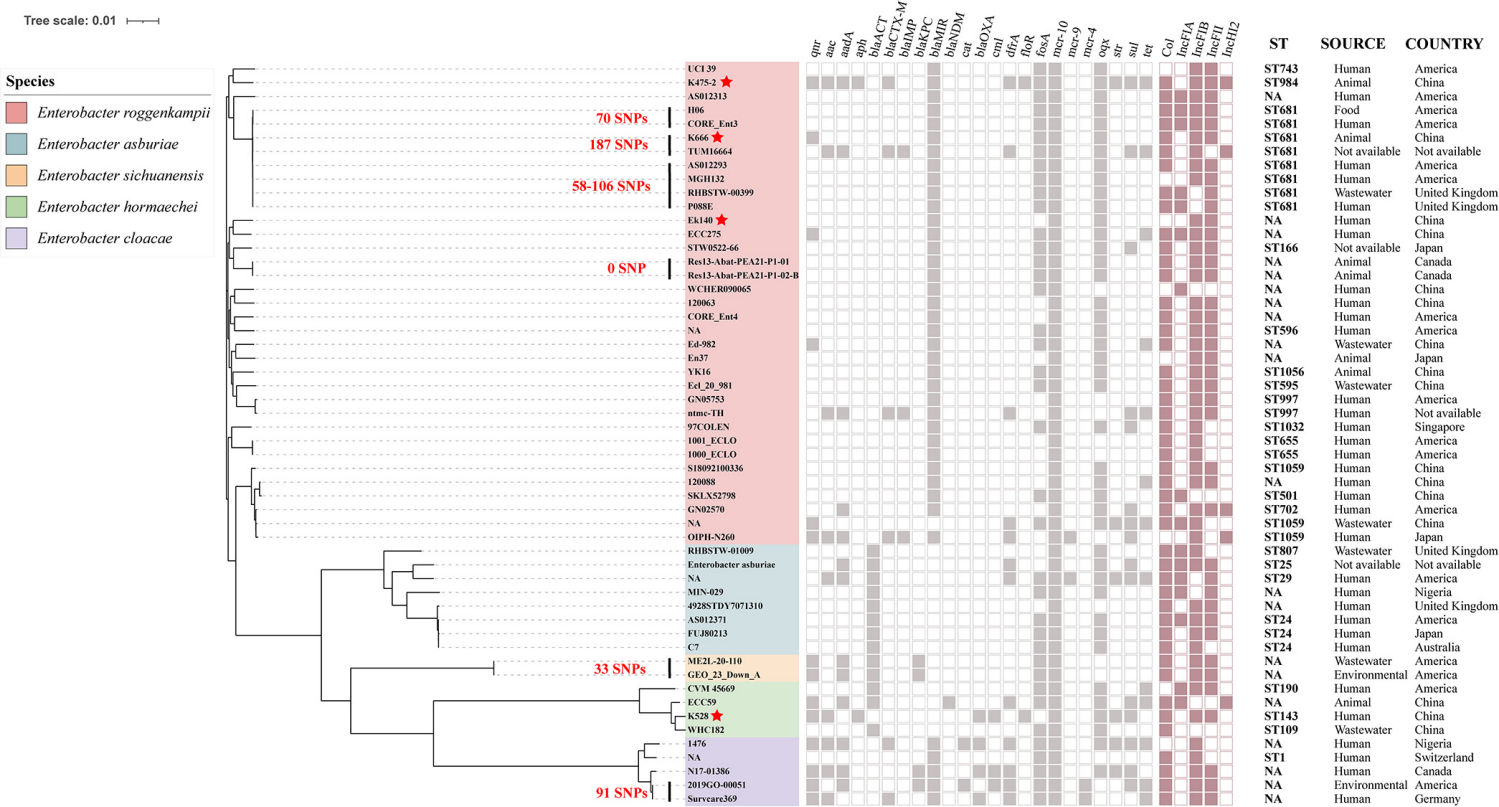
Phylogenetic tree of 54 *mcr-10*-positive Enterobacter genomes. Phylogenetic maximum-likelihood tree generated using iTOL software of SNP analysis performed using the snp-dists tool (see Table S8 in the supplemental material). The clades of *E. roggenkampii*, *E. asburiae*, *E. sichuanensis*, *E. hormaechei* and E. cloacae are highlighted in light red, blue, yellow, green and purple, respectively. A total of 4 different origins of isolates (human, animal, environmental, and wastewater) are identified.

To further investigate the prevalence and distribution of *mcr-10* in Enterobacter, we performed phylogenomic analysis based on their core genomes. A data set consisting of 54 *mcr-10*-positive Enterobacter was compiled that included the 4 strains from the current study and 50 strains from NCBI GenBank (Fig. 3 and Table S5). The *mcr-10*-bearing strains were found in 11 countries (China, America, United Kingdom, Japan, Canada, Australia, Singapore, Nigeria, Germany, Switzerland, and Vietnam) of 5 continents. These isolates were isolated from different sources, including human (feces, urine, sputum, blood, etc.), animal (chicken, dog, and pig), environment and wastewater. All strains could be assigned into 5 distinct clades, which were consistent with species classification, including E. roggenkampii (35/54 64.8%), E. asburiae (8/54 14.8%), E. sichuanensis (2/54 3.7%), E. hormaechei (4/54 7.4%) and E. cloacae (5/54 9.3%). Among them, clade E. roggenkampii was the primary lineage, containing 35 strains from human, animal, food and wastewater samples. The clade E. asburiae was the second largest lineage and contained the largest proportion of human-derived strains that included 6 of 8 strains from humans. The 54 *mcr-10-*bearing strains were positive for at least 3 categories of resistance genes, with the most prevalent being *fosA*, *oqxAB,* and *bla*_MIR-5_ (Table S6). Inc types of FIB and FII were predominant in analyzed strains.

Notably, several clonal clusters were identified from clades E. roggenkampii, E. sichuanensis, and E. cloacae. Each of these clusters contained 2 or more sources and had extremely low SNP differences (0-187 SNPs) even though they were originated from diverse geographic places, origins, or epochs (Fig. 3 and Table S8). For example, the companion animal-derived K666 and 7 strains from different origin (human, food, and wastewater) and distinct regions (America, China, and United Kingdom) were all ST681. It was worth noting that human-derived strain Survcare369 were closely related to *mcr-10*-positive strain 2019GO-00051 of environmental origin from Germany and shared a maximum difference of 91 SNPs. In addition, both strains also carried *bla*_MIR-5_ and *mcr-4.* The dissemination of *mcr-10* within companion animals, humans and other environmental sectors have posed a threat to public health in the era of ‘One Health’.

## DISCUSSION

Due to the unreasonable use of colistin in clinical treatment, animal feed, and aquaculture, the emergence of colistin-resistant bacteria has spread globally. From One Health’s perspective, animals and humans are essential components for AMR dissemination. According to previous reports, colistin resistance gene *mcr-10* has been primarily detected from human and environments, and rarely reported in animals, especially companion animals. Experimental studies have confirmed that human and pets routinely share microbiota and transient transmission of animal-derived commensal can lead to the transfer of MDR bacteria between pets and human hosts (24). Therefore, clarifying the potential transmission relationship and different characteristics of *mcr-10*-positive strains in clinical settings and their epidemiological association with companion animals-derived strains is necessary.

Here, we reported *mcr-10* in 4 ECC strains recovered from companion animals and human. *mcr-10-*harboring strain K475-2 in this study showed high MIC value (128 mg/L) for colistin compared with those previously reported (9, 25). According to earlier results on low-level colistin resistance mediated by *mcr-10*, we hypothesized that additional essential mechanisms mediate high-level resistance to colistin (9, 26, 27). Recently, Xu et al. observed that *mcr-10* expression is inducible, and it was co-upregulated with *phoPQ* to mediate the high-level colistin resistance (9). This was in line with the recent publication showing that hetero-resistance to colistin in of E. cloacae (responsible for treatment failure) was due to the expression PhoP/PhoQ (28). Besides, the mutations in TCSs that may give rise to increased colistin resistance in Enterobacter spp. were screen in both 4 colistin-resistant ECC strains (29, 30). Similar to previous research (11), there were also various variations existing in both PhoP/PhoQ, PmrA/PmrB, QseB/QseC, and MgrB, especially in PmrB, QseB, and QseC, which might activate the TCSs and modify the membrane permeability to achieve low susceptibility. Nevertheless, although these mutations might contribute to high-level colistin resistance (31), it was difficult to identify the specific amino acids involved in colistin resistance as mutations were casual and also existing in strains with intermediate MIC values (Table S2).

In previous research, as well as this study, (9, 11, 26, 27), *mcr-10*-carrying plasmids were primarily found in Enterobacter spp. strains, thus suggesting that isolates harboring *mcr-10* were stably genus-specific. Moreover, though E. cloacae and E. hormaechei were the most frequently isolated in human clinical samples, phylogenetic analysis revealed that *mcr*-*10* positive isolates were mainly distributed in the clade of E. roggenkampii (13/16, 81.25%), exhibiting significant species preference. The 3 E. roggenkampii strains in this study were assigned to different STs, indicating that the transmission of *mcr-10* among this species was clonally unrestricted. In addition, we revealed that *mcr-10* was adjacent to a XerC-type tyrosine recombinase-gene in most analyzed contigs and IS*Ec36*/IS*26* were located on the downstream of core conservative structure *xerC-mcr-10* in most strains, thus *mcr-10* might be mobilized by IS-mediated mechanisms. Furthermore, different from pMCR10_90065, *mcr-10* was first detected on a non-self-transmissible IncFIA plasmid, while the plasmid pEk140_mcr-10 was self-transmissible since a contact type IV conjugation system was located on IncFIB-FII plasmid, which likely contributed to the transmission of colistin resistance.

Besides, we also identified a close relation (SNPs < 187) between *mcr-10-*harboring isolates from human, companion animal, food, and wastewater through phylogenetic analysis. Our study revealed that the *mcr-10*-positive plasmids/ECC isolates were likely to be equipped with the cross-sector transmission ability among human, companion animals, and the environment. Further research would be essential to provide evidence to verify the potential transmission route of *mcr-10*-positive ECC between animals, humans, and the environment.

In conclusion, the present work identified the polymorphism and potential transmission of *mcr-10*-positive ECC in clinical settings, animals, and the environment. The companion animals- and human-derived strains revealed different characteristics, such as the carriage of resistance genes, and genetic environment, suggesting more target research and prevention solution needed to slow down the potential transmission between human and companion animals. Our study has filled the gap of prevalence information of *mcr-10*-positive ECC from companion animals and confirmed a real and urgent risk for *mcr-10* potential transmission between companion animals, human and their associated environments. In the future, constant monitoring of the potential transmission of *mcr-10*-positive ECC from clinical settings, companion animals, animal products and communities will be necessary to be conducted and implement tailored strategies to prevent further spread of *mcr-10*.

## MATERIALS AND METHODS

### Bacterial strains.

A total of 734 non-repetitive clinical isolates (Table S7) and 294 animal feces samples were collected from clinical samples and healthy pets in Jilin province of China in 2019. All procedures involving human samples in this study were carried out in compliance with the ethical standards of the Medical Ethic Committee of Jilin Center for Disease Control and Prevention. In total of 294 animal feces samples and 734 non-repetitive clinical isolates were spread on MacConkey Inositol Adonitol Carbenicillin Agar supplemented with 2 mg/L colistin for obtaining colistin nonsusceptibility bacteria. The *mcr-10* gene was detected by PCR and amplified products were subjected to Sanger sequencing, using primers shown in Table S1. Species of purified strains were identified by 16S rRNA gene sequencing.

### Antimicrobial susceptibility testing.

Antimicrobial susceptibility testing of *mcr-10*-bearing strains against tigecycline, ampicillin, ceftazidime, ciprofloxacin, kanamycin, meropenem, colistin, and amikacin were determined using the broth microdilution method according to Clinical and Laboratory Standards Institute (CLSI, 2021) guidelines (32) and E. coli ATCC 25922 was used as the quality control. For colistin and tigecycline, the breakpoints were interpreted by European Committee on Antimicrobial Susceptibility Testing (EUCAST, version 12.0).

### Conjugation assay.

Conjugation assay was conducted using strain E. coli C600 (Rif^R^) and E. coli J53 (Azi^R^) as recipients to investigate the transferability of *mcr*-bearing genetic elements. In brief, overnight cultures of the donor and recipient strains were mixed at the ratio of 1:4 in liquid LB medium and incubated without shaking at 37°C overnight. The mixture was then serially diluted 10 times and plated on selective media for enumerating the recipient (rifampicin/sodium azide) and transconjugant colonies (rifampicin plus colistin/sodium azide plus colistin). Transfer frequencies were calculated as the number of transconjugants per recipient cell (33). Positive transconjugants were confirmed by PCR targeting at *mcr-10*.

### Bioinformatics analysis and phylogenetic tree construction.

To analyze the genomic environment of *mcr*-positive strains, the genome of 4 isolates were extracted using the FastPure Bacteria DNA isolation minikit (Vazyme, Nanjing, China) according to the manufacturer’s instructions. The genome was extracted and sequenced by Illumina and MinION/Pacbio platform for short and long reads, respectively. The hybrid assembly of both short and long reads was preformed using Unicycler v0.4.8 (34). The genome sequences were annotated with RAST (https://rast.nmpdr.org/). Annotations of important MDR regions were further modified manually based on curated databases.

AMR genes, plasmid replicon, multilocus sequence types (STs), and mobile genetic elements were identified using ABRicate (https://github.com/tseemann/abricate). To further confirm accurate bacterial species, the ANI between *mcr*-positive isolates and reference strains of E. cloacae complex was determined by JSpeciesWS based on BLASTn (http://jspecies.ribohost.com/jspeciesws/). The BLAST Ring Image Generator (BRIG) tool was used to construct circular genome comparison map (35). Line alignment of core genetic structures were generated and visualized using EasyFig (36).

We searched for the existence of the *mcr-10* gene in sequences submitted in GenBank (https://www.ncbi.nlm.nih.gov/pathogens/, accessed 30 April 2022) with ‘mcr*’ as search keyword and filtered *mcr-10* positive genomes manually, which included draft and complete genome sequences. These genomes were re-annotated using Prokka v1.11 (37) and the core genes of the genomes were extracted and aligned using Roary v3.6.1 (38). An approximately maximum-likelihood phylogenetic tree was constructed using FastTree v1.4.3(39). Single nucleotide polymorphism (SNP) analysis was performed by snp-dists v0.7.0 for pairwise single nucleotide polymorphism distances.

### Data availability.

The complete nucleotide sequences of pK475-2_mcr-10, pK666_mcr-10, pK528_mcr-10, and pEk140_mcr-10 have been deposited in GenBank under accession numbers CP095167, CP095171, CP095178 and CP095181, respectively.
