# Supplementary material for: Emergence and Transmission of Plasmid-Mediated Mobile Colistin Resistance Gene mcr-10 in Humans and Companion Animals
Source: Microbiol Spectr. 2022 Aug 24;10(5):e02097-22. doi: 10.1128/spectrum.02097-22 (PMC9603504; doi:10.1128/spectrum.02097-22)
Supplement: Supplemental file 1 — Tables S1 and S2 and Fig. S1 and S2. Download spectrum.02097-22-s0001.pdf, PDF file, 2.0 MB [file spectrum.02097-22-s0001.pdf]

Supplementary data

Table S1. Primers used in this study.

**Primers**	**Sequences (5’-3’)***	**Product size (bp)**	**source**
mcr-10-F	CTGCTTTCCATGCTCGCTTC	957	This study
mcr-10-R	ATCCGGTGCCAGTTTGTAGG		
16s-F	AGAGTTTGATCCTGGCTCAG	1465	This study
16s-R	TACGGYTACCTTGTTACGACTT		

References

Wang, X., Wang, Y., Zhou, Y., Li, J., Yin, W., Wang, S. et al. 2018. Emergence of a novel mobile colistin resistance gene, *mcr-8*, in NDM-producing Klebsiella pneumoniae. Emerg Microbes Infec; 7: 122.

Table S2. Point mutations on chromosomal genes in the ECC isolates in this study.

Isolate	MIC(mg/L)	Mutation(s) in:						
		mgrB	*phoP*	*phoQ*	*pmrA*	*pmrB*	*qseB*	*qseC*
K528	8	V38T, A40S	-	V483L	G165S	T210S, I227L, V233T, A344T	R10K, T147P, T173A, E204D, A219V	V173I, I335V, H379N, S394T A398T, K423M, E444A
K666	8	-	-	-	-	T221A, S260A, E272D	T35N, R71H, L143Q	S92A, V179I, N338I, R432H
K475-2	128	-	-	-	-	T221A, I227V, E272D	-	D94E, Q330H, N338I
Ek140	8	-	E23V	-	-	F110Y, T221A, V266L, E272D	P147L, D183A	D94E, S183I

A

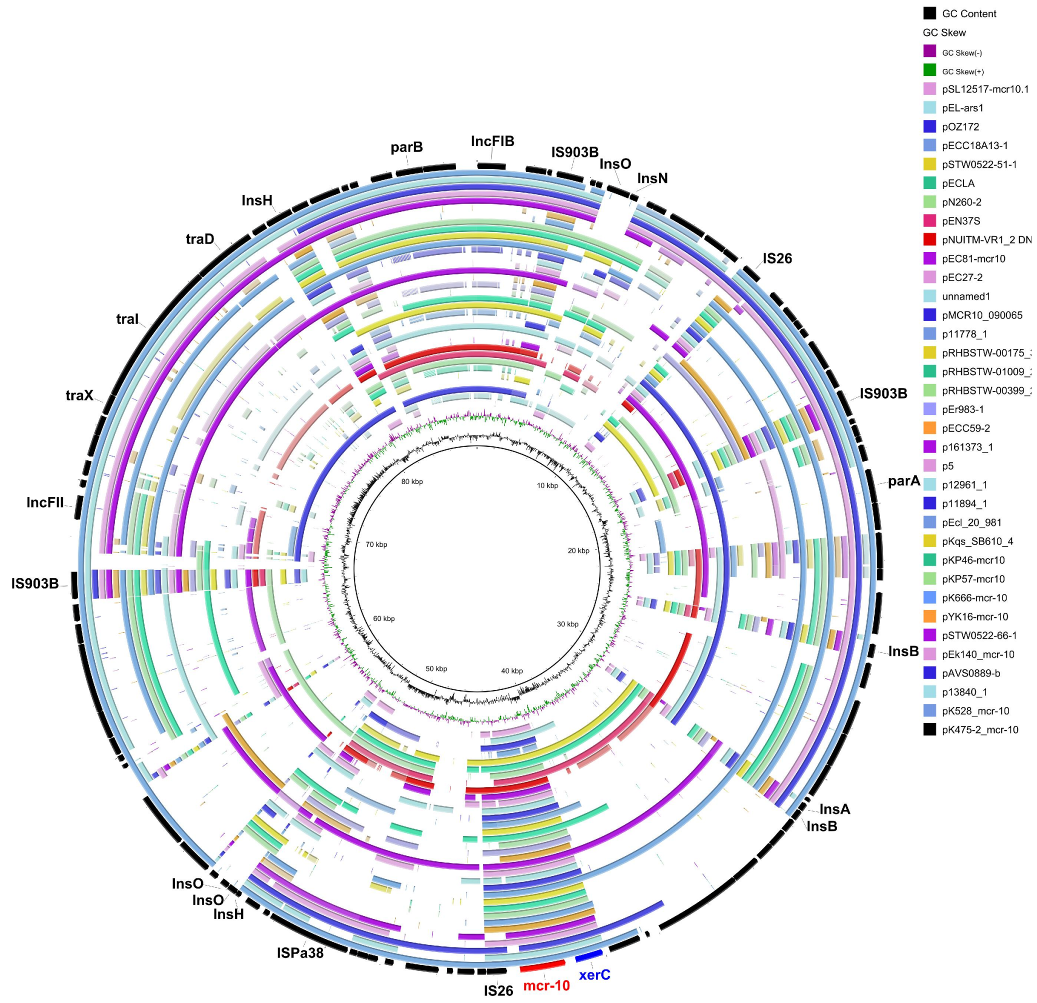

B

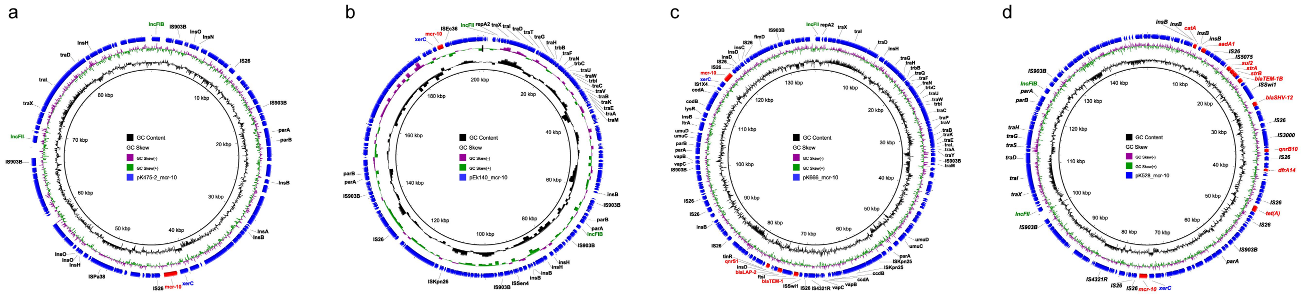

Fig S1. Genetic organization of plasmid harboring *mcr-10***.** (A) BLASTN comparison of the complement sequences of the mcr-10 plasmid found in this study and those deposited in the GenBank database. (B) Structure of plasmid carrying *mcr-10* from strains in this study. (a) Structure of plasmid pk475-2_mcr-10 from *E. roggenkampii* strain K475-2. (b) Structure of plasmid pK140_mcr-10 from *E. roggenkampii* strain Ek140. (c) Structure of plasmid pK666_mcr-10 from *E. roggenkampii* strain K666. (d) Structure of plasmid pK528_mcr-10 from *E. hormaechei* strain K528.The GC content, GC skew, nucleotide identity, and coding sequences (CDS) are all labeled in corresponding colors, and the genes are shown by the red arrow around the plasmid.

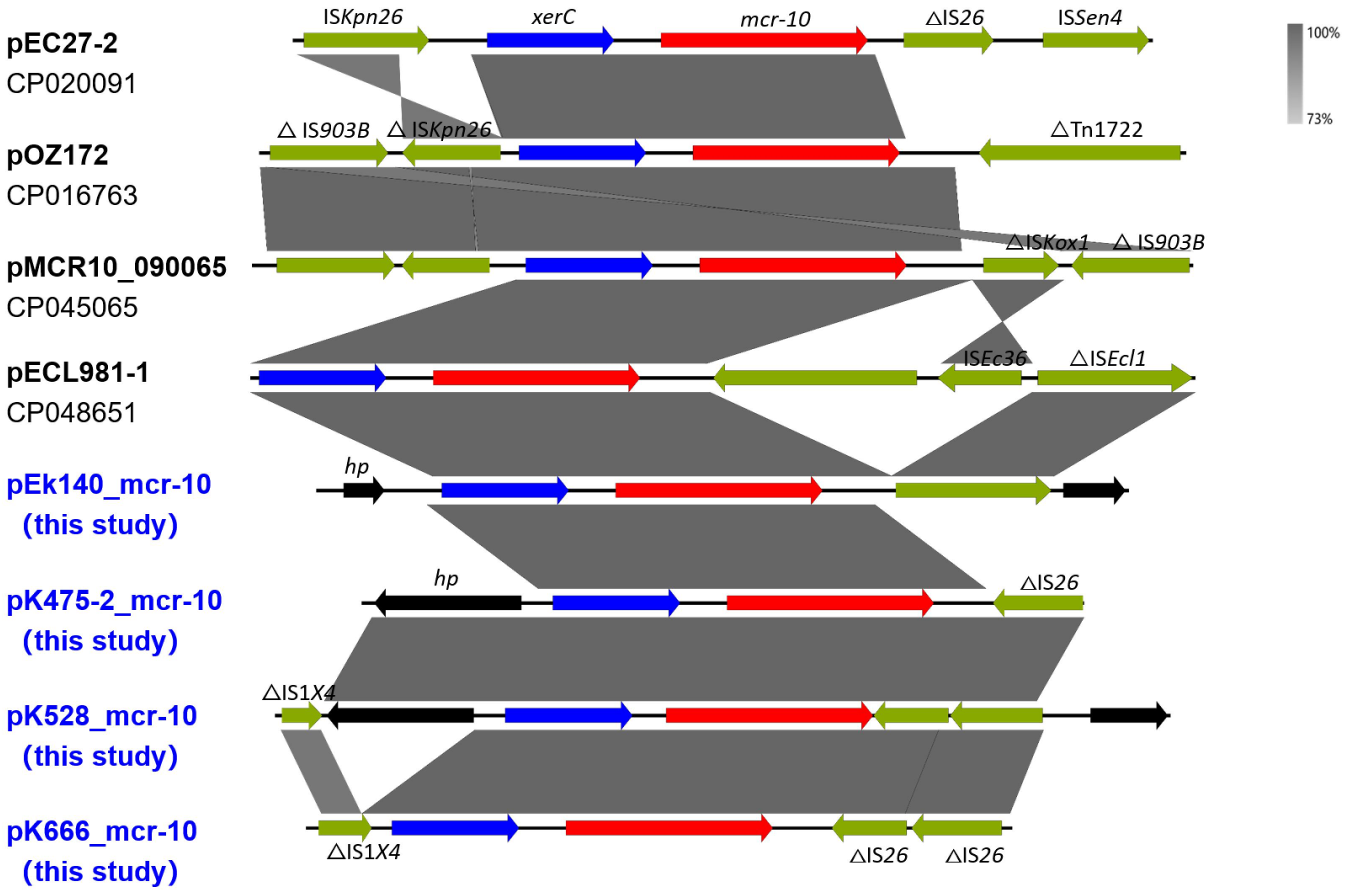

Fig S2. Comparison of four *mcr-10*-positive plasmids in the present study with different strains sharing high identity from NCBI database. ORFs with different functions are presented in various colors. ISs are shown with a green arrow. The regions with > 99% homology between different *mcr-10*-harborring strains are indicated by grey shading. △represents truncated insertion sequences or transposons.
